# *Friunavirus* Phage-Encoded Depolymerases Specific to Different Capsular Types of *Acinetobacter baumannii*

**DOI:** 10.3390/ijms24109100

**Published:** 2023-05-22

**Authors:** Olga Y. Timoshina, Anastasia A. Kasimova, Mikhail M. Shneider, Ilya O. Matyuta, Alena Y. Nikolaeva, Peter V. Evseev, Nikolay P. Arbatsky, Alexander S. Shashkov, Alexander O. Chizhov, Andrey A. Shelenkov, Yulia V. Mikhaylova, Pavel V. Slukin, Nikolay V. Volozhantsev, Konstantin M. Boyko, Yuriy A. Knirel, Konstantin A. Miroshnikov, Anastasia V. Popova

**Affiliations:** 1 Shemyakin-Ovchinnikov Institute of Bioorganic Chemistry, Russian Academy of Sciences, 117997 Moscow, Russia; lalatimosha@gmail.com (O.Y.T.); mikhailshneider@gmail.com (M.M.S.); petevseev@gmail.com (P.V.E.); kmi@bk.ru (K.A.M.); 2 State Research Center for Applied Microbiology and Biotechnology, 142279 Obolensk, Russia; xopgi@yandex.ru (P.V.S.); nikvol@obolensk.org (N.V.V.); 3 N. D. Zelinsky Institute of Organic Chemistry, Russian Academy of Sciences, 119991 Moscow, Russia; nastia-kasimova979797@mail.ru (A.A.K.); nikolay.arbatsky@gmail.com (N.P.A.); shash@ioc.ac.ru (A.S.S.); chizhov@ioc.ac.ru (A.O.C.); yknirel@gmail.com (Y.A.K.); 4 Bach Institute of Biochemistry, Research Centre of Biotechnology of the Russian Academy of Sciences, 119071 Moscow, Russia; i.matyuta@fbras.ru (I.O.M.); kmb@inbi.ras.ru (K.M.B.); 5 Center for Photonics and 2D Materials, Moscow Institute of Physics and Technology, 141700 Dolgoprudny, Russia; 6 Complex of NBICS Technologies, National Research Center “Kurchatov Institute”, 123182 Moscow, Russia; nikolaeva_ay@nrcki.ru; 7 Central Scientific Research Institute of Epidemiology, 111123 Moscow, Russia; shelenkov@cmd.su (A.A.S.); mihailova@cmd.su (Y.V.M.)

**Keywords:** bacteriophage, *Acinetobacter baumannii*, tailspike depolymerase, capsular polysaccharide, glycosidase, capsular type, crystal structure

## Abstract

*Acinetobacter baumannii* is a critical priority nosocomial pathogen that produces a variety of capsular polysaccharides (CPSs), the primary receptors for specific depolymerase-carrying phages. In this study, the tailspike depolymerases (TSDs) encoded in genomes of six novel Friunaviruses, APK09, APK14, APK16, APK86, APK127v, APK128, and one previously described *Friunavirus* phage, APK37.1, were characterized. For all TSDs, the mechanism of specific cleavage of corresponding *A. baumannii* capsular polysaccharides (CPSs) was established. The structures of oligosaccharide fragments derived from K9, K14, K16, K37/K3-v1, K86, K127, and K128 CPSs degradation by the recombinant depolymerases have been determined. The crystal structures of three of the studied TSDs were obtained. A significant reduction in mortality of *Galleria mellonella* larvae infected with *A. baumannii* of K9 capsular type was shown in the example of recombinant TSD APK09_gp48. The data obtained will provide a better understanding of the interaction of phage–bacterial host systems and will contribute to the formation of principles of rational usage of lytic phages and phage-derived enzymes as antibacterial agents.

## 1. Introduction

*Acinetobacter baumannii* is an important nosocomial pathogen listed by the World Health Organization as one of the highest-priority microorganisms for the development of new antibacterial agents [1]. In this regard, the use of virulent bacteriophages, as well as enzymes encoded in their genomes, is one of the possible approaches to control the spread of multidrug-resistant strains of *A. baumannii*.

The capsular polysaccharides (CPSs) surrounding the *A. baumannii* cells are the primary receptors for bacteriophages encoding specific structural depolymerases that determine the initial step of phage–host interaction [2,3,4]. The CPS is a high-molecular-weight carbohydrate polymer composed of repeated oligosaccharide units (K units) that are linked together by a Wzy polymerase [5,6]. Nowadays, more than 240 different gene clusters responsible for capsule biosynthesis (K loci, KL) have been identified, and each cluster is assigned a corresponding KL number [7,8,9]. 

The genus *Friunavirus* of the family *Autographiviridae*, subfamily *Beijerinckvirinae*, is the largest group of *A. baumannii* phages, which comprises over 50 phages with genomes deposited to NCBI GenBank [10]. The genomes of Friunaviruses encode only one tailspike depolymerase (TSD) specific to a certain K type or several K types with similar structures or linkages between K units [2,4]. In this group, viruses specific to K1, K2, K3-v1, K9, K19, K26, K27, K32, K37, K44, K48, K87, K89, K93, and K116 capsular types of *A. baumannii* have already been described [2,3,4,5,11,12,13].

In this work, we present the characterization and study of enzymatic activities of the TSDs encoded in the genomes of novel Friunaviruses APK09, APK14, APK16, APK86, APK127v, APK128, and previously described *Friunavirus* phage APK37.1 [11] isolated on *A. baumannii* strains belonging to K9, K14, K16, K86, K127, K128, and K37 capsular types, respectively. Three of the TSDs were also characterized by means of X-ray crystallography, revealing the structural features of these proteins. The possible antivirulence potential and therapeutic efficacy of the *Friunavirus* phage-encoded enzymes were demonstrated in the example of the TSD APK09_gp48 using a *Galleria mellonella* model. 

This work is a continuation of our previous studies on the characterization of *A. baumannii* phages encoding polysaccharide-degrading/modifying enzymes [4,5,12,14,15,16].

## 2. Results

### 2.1. Characterization of Phages Encoding the Studied Depolymerases 

Bacteriophages APK09, APK14, APK16, APK86, APK127v, and APK128 are novel bacterial viruses that were isolated from sewage and environmental (river water) samples collected in 2018 from the Moscow region in Russia on the lawns of *A. baumannii* strains B05, AB5256, D4, MAR55-66, 36-1454, and KZ-1093, which produce K9 [14], K14, K16 [17], K86 [18,19], K127 [6], and K128 [20] capsular polysaccharides (CPSs), respectively. The phages were named according to the nomenclature proposed in our previous work [4], where APK means *Acinetobacter* phage and the number of the K type to which the *A. baumannii* strain infected by the phage belongs. The designation APK127v indicates that the phage infects only the variant of K127 CPS-producing *A. baumannii* strain that carries prophage-encoded Wzy polymerase, forming the linkage between K units [6].

All the phages form clear plaques with big haloes on the host bacterial lawns, which indicates the presence of phage structural depolymerases degrading corresponding CPSs [4,12,21]. Interestingly, phage APK86 initially isolated on *A. baumannii* MAR55-66 (K86 capsular type) also infects *A. baumannii* LUH5547 (K87 capsular type), which can be explained by the fact that the depolymerase encoded in APK86 genome recognizes and degrades a similar/same linkage in CPS structures of these strains. This has already been shown for TSD of phage APK2 cleaving the same linkage in the K2 and K93 *A. baumannii* CPSs [4]. 

It has been demonstrated that the previously described *Friunavirus* phage APK37.1 also possesses a broad K-specificity and infects *A. baumannii* strains that carry KL37, KL116 CPS biosynthesis loci, and a subset of *A. baumannii* isolates that carry the KL3/KL22 with a single-base deletion in the *gtr6* gene, causing loss of the Gtr6 glycosyltransferase (capsular type designated as K3-v1) [11]. In this work, it was shown that APK37.1 was also able to infect *A. baumannii* 36-1454, the bacterial host for phage APK127v.

Phages APK09, APK14, APK16, APK86, APK127v, and APK128 have linear double-stranded DNA (dsDNA) genomes ranging in size from 41,135 to 42,013 bp and containing from 52 to 56 predicted genes located in one direction (Table 1). The genomes are flanked by direct terminal repeats (DTRs) of 357–428 bp in length. The GC content of the genomes is 39.2–39.4% and is close to the typical GC content of *A. baumannii* genomes [22]. No tRNA genes were identified.

The APK37.1 linear dsDNA genome was annotated and described previously [11]. 

**Figure 1:**
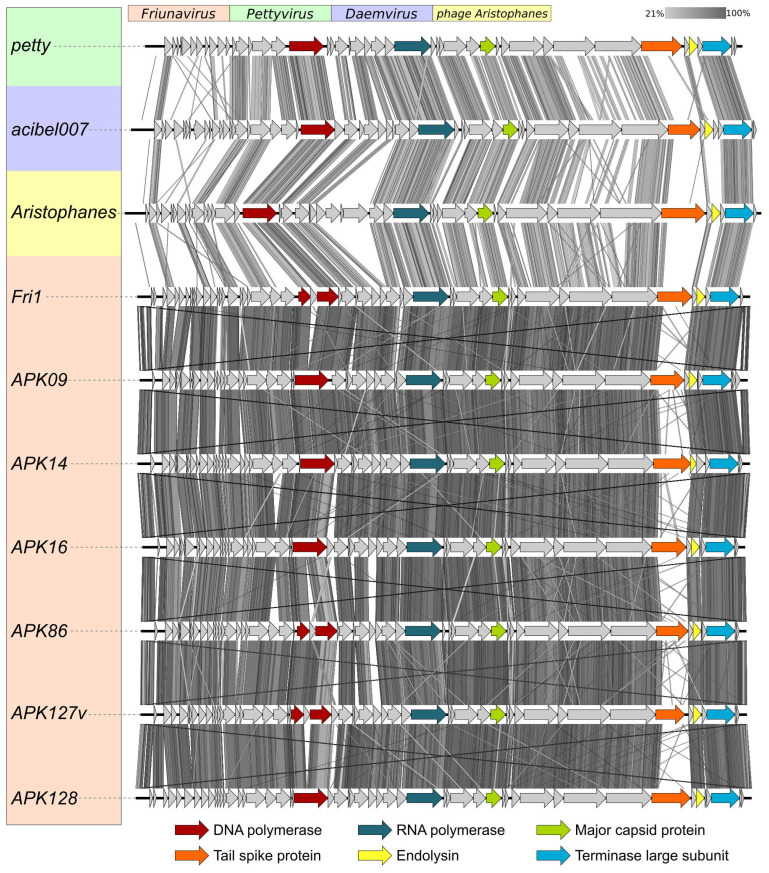
Comparison of the genome sequences of phages APK09, APK14, APK16, APK86, APK127v, APK128, and genomes of other representatives of *Beijerinckvirinae* subfamily infecting *A. baumannii*. The percentage of sequence similarity is indicated by the intensity of the gray color shown in the legend in the upper right corner. Vertical blocks between analyzed sequences indicate regions with at least 21% similarity.

Novel phages APK09, APK14, APK16, APK86, APK127v, and APK128 shared similar genome organization with the phages of the genus *Friunavirus* of the subfamily *Beijerinckvirinae*, family *Autographiviridae* (Figure 1). The genome comparison revealed that the most variable regions fall in the central and 3′-parts of the genes encoding TSDs responsible for recognizing and cleaving different *A. baumannii* CPSs and, in the early gene regions, essential for the first steps of phage infection [23,24]. It is notable that the putative DNA polymerase protein of phages APK127v and APK86 is encoded by two parts split by the HNH homing endonuclease gene. 

**Figure 2:**
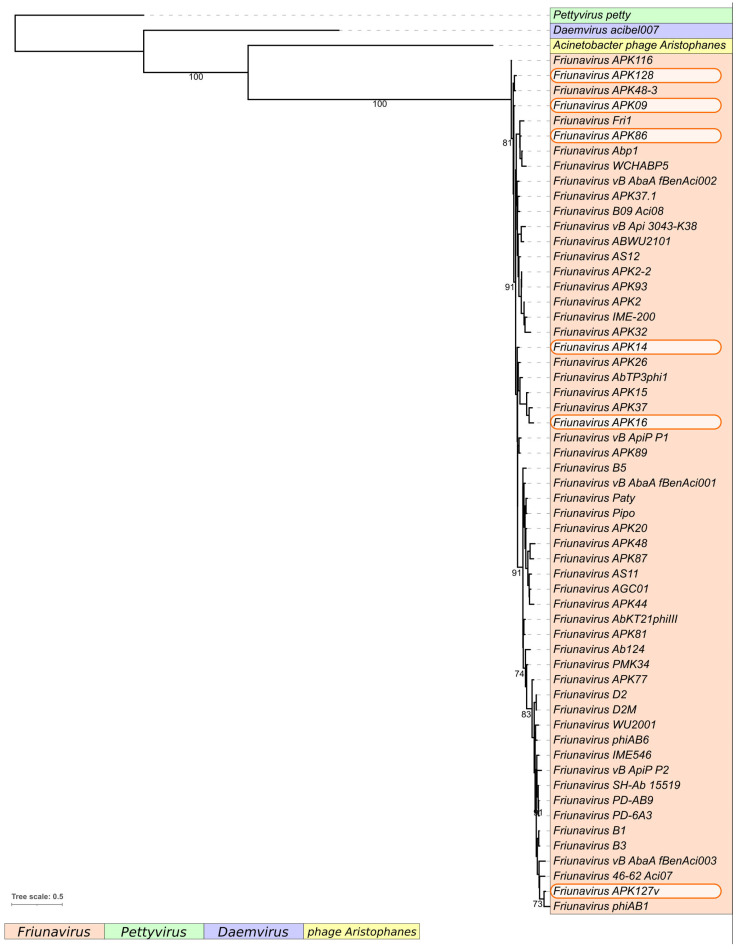
Best-scoring tree obtained with RAxML-NG based on concatenated amino acid sequences of major capsid protein, large subunit of terminase, head-to-tail connector protein, DNA polymerase, and RNA polymerase encoded in the genomes of 60 *Beijerinckvirinae* phages. Percentage of bootstrap support is shown near corresponding branches. The scale bar shows 0.5 estimated substitutions per site. Pettyvirus petty was used as an outgroup.

The comparison of nucleotide intergenomic similarity (Figure S1) and the results of the phylogenetic analysis of conservative proteins (Figure 2) also clearly indicated that the novel *A. baumannii* phages belong to the genus *Friunavirus*.

### 2.2. Phage Tailspike Depolymerases

Like in other described phages of the genus *Friunavirus* [2,4,12], TSDs of novel phages APK09, APK14, APK16, APK86, APK127v, and APK128 and previously described phage APK37.1 [11] were formed by single proteins encoded by the genes located at the end of structural modules of the phage genomes (Figure 1).

At the amino acid level, all studied TSDs share the highest degree of similarity in their N-terminal domains (approximately the first 140–150 amino acids of the proteins) responsible for the attachment of variable CPS-recognizing/degrading parts of the tailspikes to the phage particles. At the same time, CPS-recognizing/degrading parts, which determine the K specificity of Friunaviruses, differ significantly from each other.

According to BLASTp analysis, TSD APK09_gp48 was almost identical to the protein encoded by another Friunavirus vB_AbaP_B1 (gp45; GenBank accession number: YP_009610331) isolated in Portugal [2]. The bacterial hosts of both phages belong to the same K9 capsular type. 

Noteworthy, Friunavirus APK09 and previously described Myovirus AM24 [14] were isolated and propagated on the same bacterial host, *A. baumannii* B05. This means that TSDs encoded in APK09 and AM24 genomes can specifically recognize and degrade the CPS of the same structure at the initial step of phage–host interaction. However, TSD APK09_gp48 does not share a high level of similarity with TSD AM24_gp50 (APD20249, the coverage obtained to an E-value of 8 × 10^−40^ was 76%, with an identity of 28.57%) at the amino acid level. Moreover, APK09_gp48 differs from TSD gp47 of Myovirus BS46 (QEP53229, the coverage obtained to an E-value of 3 × 10^−37^ was 79% with an identity of 27.79%) isolated on the bacterial lawn of *A. baumannii* AC54 [25] also having K9 CPS structure [26]. The lack of homology between N-terminal parts of APK09_gp48 and TSDs of Myoviruses AM24 and BS46 can be explained by the fact that these structurally conserved parts of the proteins are very different in bacteriophages representing distant taxonomic groups. Despite the low level of similarity of APK09_gp48 with AM24_gp50 at the amino acid level, the CPS-recognizing/degrading parts of these proteins share the structural similarity according to HHpred analysis.

BLASTp analysis revealed that the closest homologs of TSD APK14_gp49 were tailspike protein of *Acinetobacter* phage AB_SZ6 (URQ05102, the coverage obtained to an E-value of 0 was 100% with an identity of 90.36%), which also belongs to the genus *Friunavirus* and protein of *Acinetobacter* phage MD-2021a (CAH1066870, the coverage obtained to an E-value of 0 was 79% with an identity of 66.96%). The similarity of amino acid sequences of CPS-recognizing/degrading parts of APK14_gp49 with the listed above proteins indicates that they, most likely, specifically interact with CPS of the same structure.

CPS-recognizing/degrading part of APK16_gp47 has no homologs among phage proteins deposited in GenBank but shares amino acid similarity with hypothetical protein (WP_228157370) encoded in *A. baumannii* genome.

Analysis of the similarity of APK37.1_gp49 with the other proteins deposited to Genbank has already been performed [11]. The TSD is homologous to TSD gp44 (AZU99445) of Friunavirus vB_AbaP_APK37 described in our previous work [4] and gp48 of Friunavirus AbTP3phi1 (UNI74976). The bacterial hosts of phages APK37 and vB_AbaP_APK37, *A. baumannii* KZ-1101, and NIPH146, respectively, belong to the same K37 capsular type. 

Interestingly, TSD APK86_gp49 was almost identical to TSD APK87_gp48 (QGK90498) of previously characterized phage vB_AbaP_APK87 [4]. This means that these depolymerases are likely specific to both K86 and K87 CPSs.

According to BLASTp analysis, CPS-recognizing/degrading part of APK127v_gp47 was homologous to the corresponding parts of the protein of *Acinetobacter* Myovirus vB_AbaM_IME512 (AYP69084, the coverage obtained to an E-value of 1 × 10^−168^ was 76% with an identity of 54.01%) and the protein of *Acinetobacter* Myovirus WCHABP1 (YP_009604496, the coverage obtained to an E-value of 1 × 10^−160^ was 76% with an identity of 52.33%).

APK128_gp45 was almost identical to the protein gp41 of Friunavirus phiAB1 (YP_009189380), indicating, most likely, the same K specificity of phages APK128 and phiAB1. APK128_gp45 also shares some amino acid similarities with TSD AS12_gp42 of Friunavirus vB_AbaP_AS12 (YP_009599229), specific to K19 capsular type [12], and TSD APK116_gp43 of Friunavirus vB_AbaP_APK116 (QHS01530), specific to K116 capsular type [4].

According to HHpred analysis, the amino acid sequences of all TSDs had the pectate lyase 3 and Glyco_hydro_28 conserved Pfam motifs. The N-terminal parts of APK09_gp48, APK14_gp49, APK16_gp47, APK37.1_gp49, APK86_gp49, APK127v_gp47, and APK128_gp45 share structural similarity with the N-terminal part of *Escherichia* bacteriophage T7 tail fiber protein (PDB ID: 7EY9). The remaining parts of all analyzed TSDs contain the regions which show structural similarities with different phage tailspikes.

APK09_gp48, APK14_gp49, APK16_gp47, APK37.1_gp49, APK86_gp49, APK127v_gp47, APK128_gp45 are 760-, 851-, 785-, 824-, 720-, 663-, and 878-amino-acid proteins with predicted molecular weights of 82.52 kDa, 95.4 kDa, 84.42 kDa, 90.81 kDa, 77.83 kDa, 71.87 kDa, and 96.94 2 kDa, respectively. 

**Figure 3:**
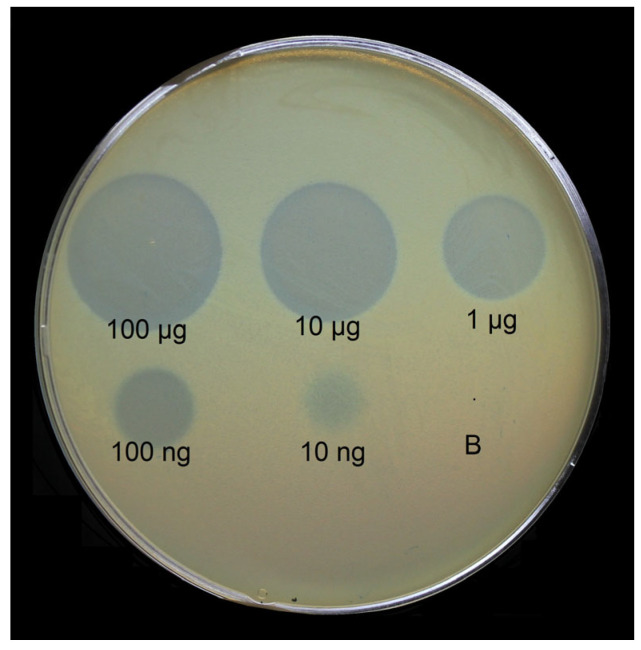
Spot test with serial 10-fold titration of purified recombinant TSD APK09_gp48 on *A. baumannii* B05 lawn after 16 h of incubation. B—buffer for storage of the protein as a negative control.

In order to avoid possible TSD aggregation due to the hydrophobicity of their N-termini, the latter were deleted, yielding fragments corresponding to only CPS-recognizing/degrading parts listed in Table S1. The TSD genes without regions responsible for particle-binding N-terminal domains were cloned, expressed, and purified by immobilized metal ion affinity chromatography, followed by ion-exchange chromatography. The enzymatic activity of N-deletion mutants of different *A. baumannii*-phage TSDs toward corresponding CPSs has already been demonstrated in our previous works [4,12,14,15]. An example of serial 10-fold titration of one of the purified recombinant N-deletion TSD mutants (hereinafter, recombinant TSD) on the bacterial lawn of a host strain after 16 h of incubation is presented in Figure 3.

### 2.3. Mechanism of Cleavage of A. baumannii CPSs by Specific Phage TSDs 

To elucidate the mechanisms of depolymerase action, the purified CPSs of *A. baumannii* host strains B05 (K9 capsular type), AB5256 (K14), D4 (K16), MAR55-66 (K86), 36-1454 (K127), and KZ-1093 (K128) were cleaved with recombinant TSDs APK09_gp48, APK14_gp49, APK16_gp47, APK86_gp49, APK127v_gp47, and APK128_gp45, respectively. Considering that phage APK37.1 infects *A. baumannii* strains assigned to several different K types, the enzymatic activity of TSD APK37.1_gp49 was studied toward CPSs of *A. baumannii* strain KZ-1101 (K37) and AB5001 (K3-v1). 

The resulting oligosaccharide products were fractionated by Fractogel TSK HW-40S gel permeation chromatography. Their structures were established by one- and two-dimensional ^1^H and ^13^C NMR spectroscopy and were confirmed by high-resolution electrospray ionization mass spectrometry (HR ESI-MS) (Table S2).

**Figure 4:**
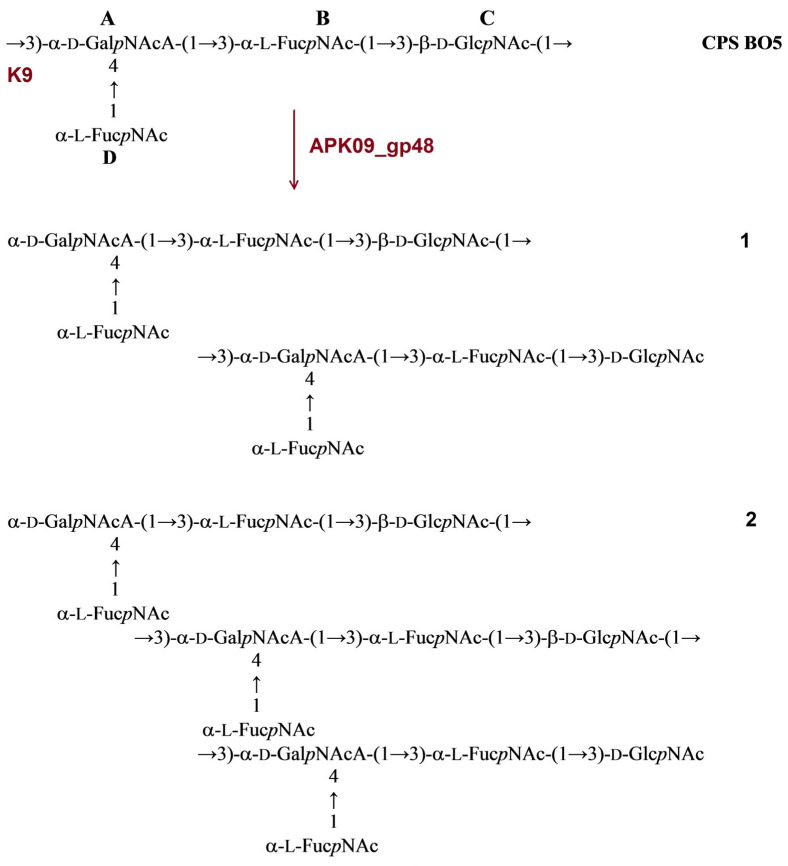
Cleavage of the K9 CPS of *A. baumannii* B05 with recombinant TSD APK09_gp48, giving rise to oligosaccharides **1** and **2** corresponding to dimer (**1**) and trimer (**2**) of the K unit, respectively.

**Figure 5:**
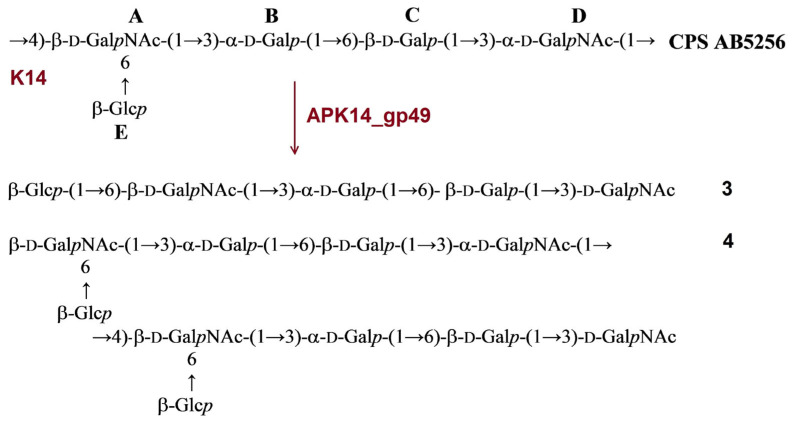
Cleavage of the K14 CPS of *A. baumannii* AB5256 with recombinant TSD APK14_gp49, giving rise to oligosaccharides **3** and **4** corresponding to a monomer and a dimer of the K unit, respectively.

**Figure 6:**
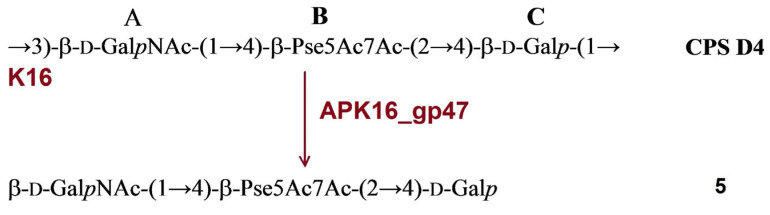
Cleavage of the K16 CPS of *A. baumannii* D4 with recombinant TSD APK16_gp47, giving rise to oligosaccharide **5** corresponding to monomer of the K unit.

**Figure 7:**
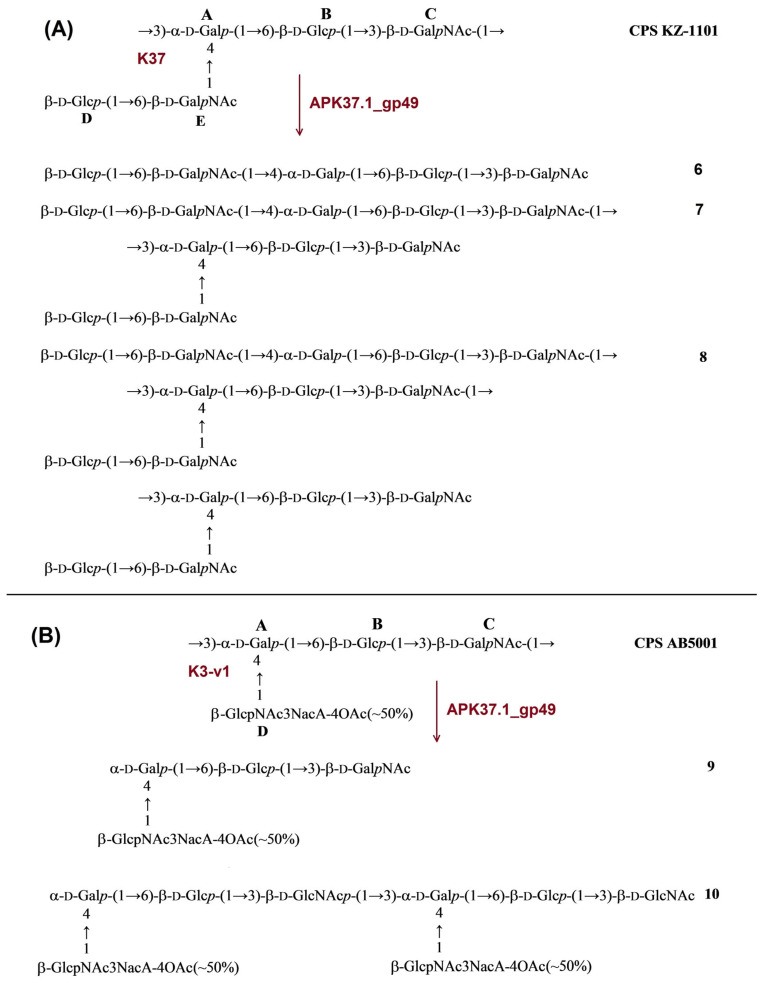
(**A**) Cleavage of the K37 CPS of *A. baumannii* KZ-1101 with recombinant TSD APK37.1_gp49, giving rise to oligosaccharides **6**-**8** corresponding to monomer (**6**), dimer (**7**), and trimer (**8**) of the K unit, respectively. (**B**) Cleavage of K3-v1 CPS of *A. baumannii* AB5001 with recombinant TSD APK37.1_gp49, giving rise to oligosaccharides **9** and **10** corresponding to monomer (**9**) and dimer (**10**) of the K unit, respectively.

**Figure 8:**
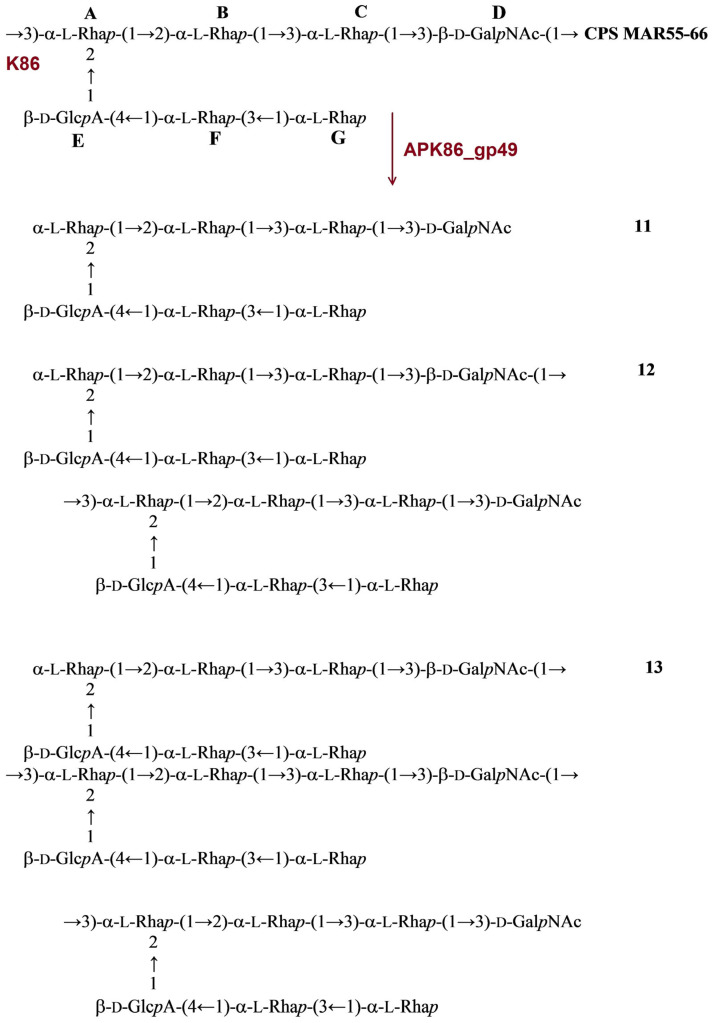
Cleavage of the K86 CPS of *A. baumannii* MAR55-66 with recombinant TSD APK86_gp49, giving rise to oligosaccharides **11**–**13** corresponding to monomer (**11**), dimer (**12**), and trimer (**13**) of the K unit, respectively.

**Figure 9:**
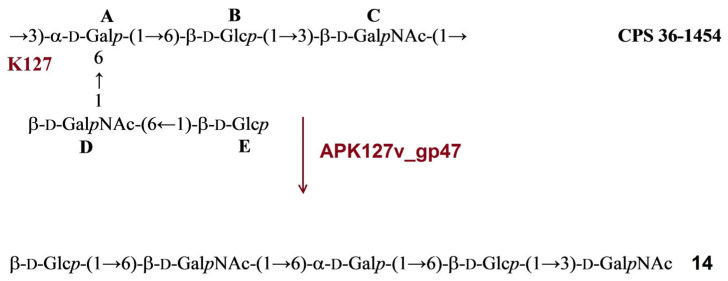
Cleavage of the K127 CPS of *A. baumannii* 36-1454 with recombinant TSD APK127v_gp47, giving rise to oligosaccharide **14** corresponding to monomer of the K unit.

**Figure 10:**
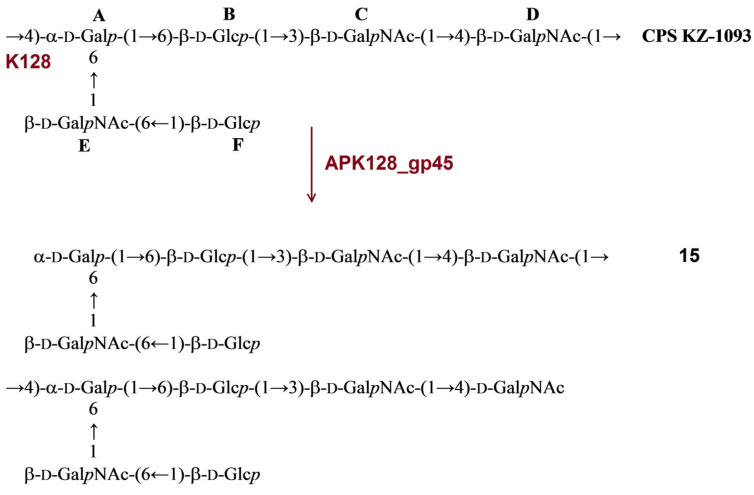
Cleavage of the K128 CPS of *A. baumannii* KZ-1093 with recombinant TSD APK128_gp45, giving rise to oligosaccharide **15** corresponding to dimer of the K unit.

All oligosaccharides had the same monosaccharide composition as the CPSs they were derived from. The ^1^H and ^13^C NMR spectra of the oligosaccharides that corresponded to the K unit monomers were fully assigned by two-dimensional shift-correlated experiments (^1^H-^1^H correlation spectroscopy [COSY], ^1^H-^1^H total correlation spectroscopy [TOCSY], and ^1^H-^13^C heteronuclear single-quantum coherence [HSQC] spectroscopy) and compared with the data of the corresponding CPSs. Linkage and sequence analyses by two-dimensional ^1^H-^1^H rotating-frame nuclear Overhauser effect (ROESY) and ^1^H-^13^C heteronuclear multiple-bond correlation (HMBC) experiments enabled elucidation of full structures of the oligosaccharides, as shown in Figure 4, Figure 5, Figure 6, Figure 7, Figure 8, Figure 9 and Figure 10. 

The ^13^C NMR chemical shifts of all but two monosaccharide residues in the K unit monomers were essentially the same in the oligosaccharides and the corresponding CPSs, whereas those of the residues at the reducing and nonreducing ends of the oligosaccharides were different. On this basis, the glycosidic linkages that were cleaved in the K9, K14, K16, K37/K3-v1, K86, K127, and K128 CPSs by recombinant TSDs APK09_gp48, APK14_gp49, APK16_gp47, APK37.1_gp49, APK86_gp49, APK127v_gp47, APK128_gp45, respectively, could be identified (Table 2).

As expected, the monosaccharides that occupy the reducing end of the oligosaccharides (d-GlcNAc in **1**, **2**, **12**, **13**, and **15**, d-GalNAc in **3**, **4**, **6**-**10**, **14**, and **15**, d-Gal in **5**) were present in two anomeric forms (α and β), which showed in the ^13^C NMR spectrum the C-1 signals characteristic for nonlinked monosaccharides at δ 92.3–92.5 for α anomers and δ 95.7–96.5 for β anomers. Significant upfield displacements (by 7.3 to 10.2 ppm) were observed for the signals for C-4 of units **A** and **C** in oligosaccharides derived by cleavage of the CPSs of *A. baumannii* AB5256 and KZ1093, and the signals for C-3 of units **A** and **D** in oligosaccharides from the CPSs of *A. baumannii* B05, D4, KZ1101, AB5001, 36-1454 and MAR55-66 (Figure 4, Figure 5, Figure 6, Figure 7, Figure 8, Figure 9 and Figure 10). Therefore, these carbons that were linked in the CPSs became nonlinked in the oligosaccharides. These data defined the structures of the oligosaccharides obtained by depolymerization of the CPSs (Figure 4, Figure 5, Figure 6, Figure 7, Figure 8, Figure 9 and Figure 10) and, as a result, identified the linkages that were cleaved by phage TSDs (Table 2).

#### 2.3.1. Cleavage of the *A. baumannii* B09 CPS by Recombinant TSD APK09_gp48

The structure of CPS produced by *A. baumannii* B05 was identical to the structure of *A. baumannii* MDR_TJ assigned to the K9 capsular type [27,28]. The CPS of strain B05 has a tetrasaccharide K unit, which contains one residue each of α-d-Gal*p*NAcA (unit **A**) and β-d-Glc*p*NAc (unit **C**) and two residues of 2-acetamido-3,6-dideoxy-D-galactose (α-Fuc*p*NAc, units **B** and **D**). No K unit monomer, but dimer **1** and trimer **2** were obtained from the B05 CPS upon cleavage by depolymerase APK09_gp48 (Figure 4).

#### 2.3.2. Cleavage of the *A. baumannii* AB5256 CPS by Recombinant TSD APK14_gp49

The CPS of *A. baumannii* AB5256 was found to be identical to the K14 CPS of *A. baumannii* O11 [29] and D46 [30]. It has a branched pentasaccharide K unit containing one residue each of α-d-Gal*p*NAc (unit **A**), β-d-Gal*p* (unit **B**), α-d-Gal*p* (unit **C**), β-d-Gal*p*NAc (unit **D**), and β-d-Glc*p* (unit **E**). Cleavage of the *A. baumannii* AB5256 CPS with depolymerase APK14_gp49 resulted in monomer **3** and dimer **4** of the K unit.

#### 2.3.3. Cleavage of the *A. baumannii* D4 CPS by Recombinant TSD APK16_gp47

The structure of *A. baumannii* D4 was established earlier [17]. It has a trisaccharide K-unit containing common monosaccharides β-d-Gal*p*NAc (unit **A**) and β-d-Gal*p* (unit **C**). The K16 CPS also includes a di-N-acetyl derivative of a higher aldulosonic acid, namely, 5,7-diamino-3,5,7,9-tetradeoxy-l-*glycero*-l-*manno*-non-2-ulosonic (pseudaminic) acid (Pse) (unit **B**). Treatment of the CPS with depolymerase APK16_gp47 resulted only in monomer (**5**) of the K unit (Figure 6).

#### 2.3.4. Cleavage of the *A. baumannii* KZ-1101 and AB5001 CPSs by Recombinant TSD APK37.1_gp49

The CPS of *A. baumannii* KZ-1101 was identical to the K37 CPS of *A. baumannii* NIPH146 [31]. It has a pentasaccharide K unit containing common monosaccharides α-d-Gal*p* (unit **A**), β-d-Glc*p* (units **B** and **D**), and β-d-Gal*p*NAc (units **C** and **E**). Upon cleavage with TSD APK37.1_gp49, the CPS of KZ1101 gave oligosaccharides **6**-**8** corresponding to monomer (**6**), dimer (**7**), and trimer (**8**) of the K unit (Figure 7A). 

The structure of the CPS of *A. baumannii* AB5001 assigned to the K3-v1 type was determined earlier [11]. The CPS has a branched tetrasaccharide repeating unit containing one residue each of α-d-Gal*p* (unit **A**), β-d-Glc*p* (unit **B**), β-d-Gal*p*NAc (unit **C**), and β-d-Glc*p*NAc3Ac (unit **D**). Depolymerization of the CPS with TSD APK37.1_gp49 led to monomer (**9**) and dimer (**10**) of the K unit (Figure 7B).

#### 2.3.5. Cleavage of the *A. baumannii* MAR55-66 CPS by Recombinant TSD APK86_gp49

The structure of the *A. baumannii* MAR55-66 CPS was established earlier [18,19]. The CPS of MAR55-66 is distinguished by the presence of β-d-Glc*p*A (unit **E**), β-d-Gal*p*NAc (unit **D**), and five residues of α-L-rhamnose (α-L-Rha*p*, units **A**, **B**, **C**, **F**, **G**) in a heptasaccharide K unit. Treatment of the CPS with depolymerase APK86_gp49 resulted in monomer (**11**), dimer (**12**), and trimer (**13**) of the repeating unit (Figure 8).

#### 2.3.6. Cleavage of the *A. baumannii* 36-1454 CPS by Recombinant TSD APK127v_gp47

The structure of the *A. baumannii* 36-1454 CPS has been established recently [6]. The CPS is built up of pentasaccharide K unit containing common monosaccharides β-d-Glc*p* (units **B** and **E**), β-d-Gal*p*NAc (units **C** and **D**), and α-d-Gal*p* (unit **A**). Treatment of the CPS with depolymerase APK127v_gp47 resulted in only monomer (**14**) of the K unit (Figure 9).

#### 2.3.7. Cleavage of the *A. baumannii* KZ-1093 CPS by Recombinant TSD APK128_gp45

The structure of the CPS of *A. baumannii* KZ-1093 was established earlier [20]. It has a branched hexasaccharide repeating unit composed of α-d-Gal*p* (unit **A**)**,** β-d-Glc*p* (units **B** and **F**), and β-d-Glc*p*NAc (units **C** and **E**). Upon cleavage with depolymerase APK128_gp45, the CPS gave a single oligosaccharide (**15**), which corresponded to a K unit dimer (Figure 10).

The data obtained indicated that oligosaccharides **1**–**15** were derived from the CPSs by specific hydrolytic cleavage of a linkage between the K units. Therefore, depolymerases APK09_gp48, APK14_gp49, APK16_gp47, APK37.1_gp49, APK86_gp49, APK127v_gp47, and APK128_gp45 are glycosidases that cleave the certain linkages (Table 2) in the CPSs of *A. baumannii* B05, AB5256, D4, KZ-1101/AB5001, MAR55-66, 36-1454, and KZ-1093, respectively. 

### 2.4. The Structure of the TSDs

**Figure 11:**
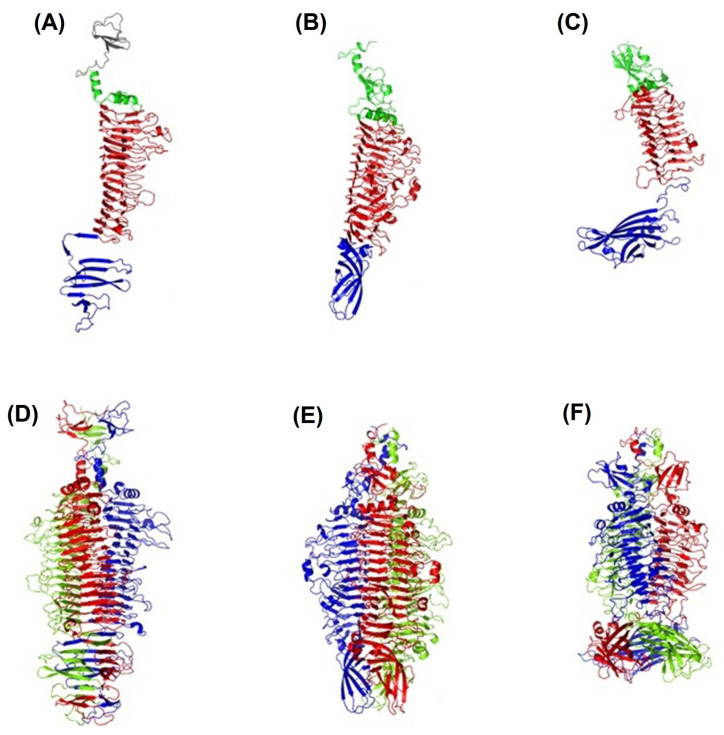
Crystal structures of the TSDs. Monomers of APK16_gp47 (**A**), APK14_gp49 (**B**), and APK09_gp48 (**C**) from the corresponding asymmetric units are in similar orientation and colored by domains: N-terminal—green; central β-helix domain—red; and C-terminal—blue. Part of the particle-binding domain of APK16_gp47 is colored gray. Crystallographic trimers of APK16_gp47 (**D**), APK14_gp49 (**E**), and APK09_gp48 (**F**) are colored by chains and are similarly oriented.

Three different TSDs, APK09_gp48, APK14_gp49, and APK16_gp47, have been chosen for structural studies. All three proteins displayed a common depolymerase three-domain architecture (Figure 11A–C). The asymmetric unit of each studied TSD contains one protein chain, but contact analysis demonstrated that these proteins form common trimers in a crystal (Figure 11D–F).

#### 2.4.1. Crystal Structure of TSD APK16_gp47 

In the case of APK16_gp47, the protein includes a part of N-terminal particle-binding domain (residues 154–213), which has three antiparallel β-strands, the following N-terminal helix-turn-helix domain (214–252), a large central right-handed β-helix domain (253–661) having 15 rungs, as well as a C-terminal seven-stranded β-sandwich domain (662–785) (Figure 11A). 

Rungs 7–9 (numbering starts from N-terminus) of the β-helix domain are complete rungs and are formed by three β-strands B1, B2, and B3, separated by turns T1, T2, and T3, where B1, B3, and T3 are solvent-exposed, and T2 faces the three-fold symmetry axis of the trimer. Rungs 1–3 have only two β-strands, B2 and B3, while the B1 region represents a loop. Rungs 4, 14, and 15 lack B2 and possess only B1 and B3. Finally, rungs 5–6 and 10–13 have B1 and a long β-strand combining B2 and B3. Besides β-sheet hydrogen bonding, the β-helix domain is additionally fastened by 18 salt bridges and hydrophobic interactions between inward-facing stacks of side chains of valine, leucine, isoleucine, and phenylalanine. Noteworthy, none of the pairs of stacked cysteines C348, C381, C413, and C434 form disulfide bonds, in spite of their location proximity.

APK16_gp47 crystallographic trimer has a diameter of 75Å and a length of about 150Å (without N-terminal particle-binding domain) (Figure 11D). Each subunit of the trimer has an interface area of about 4928Å2 (16% of the total subunit area). The trimer is stabilized by a total of 201 hydrogen bonds, 27 salt bridges and hydrophobic interactions, mainly between the β-helix domains of three subunits (formed by the side chains of L226, F253, I259, V279, I591, I614, I621, I643, and V650) and between the β-sandwich domain of three subunits (formed by the side chains of I689, I708, L722, L724, I728, V741, I746, and V748). The trimer has two cavities—a small one in the N-terminal domain and a large protruding cavity in the central β-helix domains, which, however, lack solvent molecules.

#### 2.4.2. Crystal Structure of TSD APK14_gp49 

APK14_gp49 structure consists of an N-terminal domain (residues 153–277) that possesses two α-helices (167–173 and 261–274) as well as four stranded β-sheet (176–241), a central β-helix domain (278–730) having 14 rungs and a C-terminal six-stranded β-barrel domain (731–851) (Figure 11B).

Rungs 6–12 of the central β-helix domain are rungs, where B1, B3, and T3 are solvent-exposed, and T2 faces the three-fold symmetry axis of the trimer. Rungs 1–5 have only two β-strands, B2 and B3, and rungs 13–14 lack B2 and possess only B1 and B3. The APK14_gp49 β-helix domain is stabilized by 10 salt bridges, 79 hydrogen bonds, and hydrophobic interactions in a manner similar to those of APK16_gp47.

APK14_gp49 crystallographic trimer has a diameter of 75Å and a length of about 150Å (Figure 11E). Each subunit of the trimer has an interface area of about 2948Å^2^ (11% of the total subunit area). The trimer is dominantly stabilized by polar interactions, including a total of 105 hydrogen bonds and 15 salt bridges, while the hydrophobic impact is relatively small. The trimer has two non-interacting cavities in the central β-helix domain and one cavity in the N-terminal domain. In contrast to TSD APK16_gp47, in the APK14_gp49 trimer, the central cavities contain solvent molecules. 

#### 2.4.3. Crystal Structure of TSD APK09_gp48

APK09_gp48 structure has an N-terminal four-stranded β-sheet domain (residues 155-270), a central β-helix domain (271–568) having 11 rungs, as well as a C-terminal nine-stranded β-barrel domain (569–760) (Figure 11C). The latter domain of APK09_gp48 is about to be perpendicular to the long axis of the central domain, which is in contrast to APK14_gp49, where these domains are almost aligned along the same axis.

Each of the rungs 2, 3, 7–9 of the central β-helix domain have three β-strands (B1–B3), where B1, B3, and T3 are solvent-exposed, and T2 faces the three-fold symmetry axis of the trimer. Rungs 1 and 4 have only two β-strands, B2 and B3, rung 10 lacks B3, and rung 11 lacks both B1 and B3. Finally, rungs 5 and 6 have four strands, B1–B4, with B3 and T3 being faced toward the three-fold symmetry axis. The β-helix domain is stabilized by 26 salt bridges, 116 hydrogen bonds, and hydrophobic interactions in a manner similar to those of APK16_gp47 and APK14_gp49.

APK09_gp48 crystallographic trimer has a diameter of 65Å and a length of about 140Å (Figure 11F). Each subunit of the trimer has an interface area of about 4462Å^2^ (17% of the total subunit area). The trimer is dominantly stabilized by polar interactions, including a total of 204 hydrogen bonds, 36 salt bridges, and hydrophobic interactions via side chains of V504, V544, I546, L562, and I564. The trimer has one cavity in the central β-helix domain. However, the moderate resolution did not allow us to model most of the solvent molecules in the APK09_gp48 structure, including the intra-trimer cavity.

**Figure 12:**
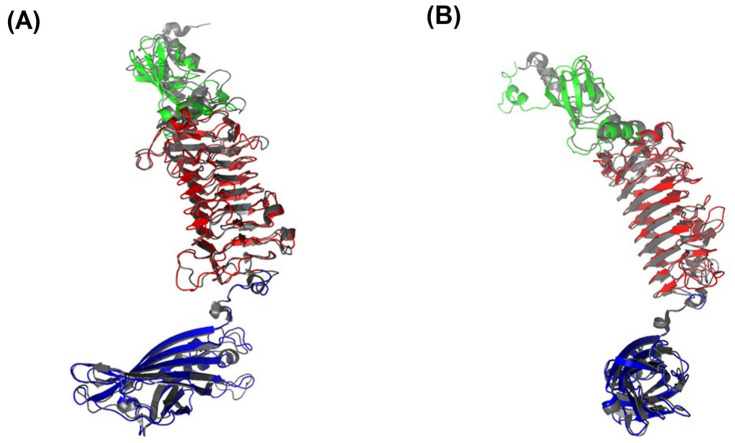
Superposition of monomers of TSD APK09_gp48 (colored as in Figure 11C) (**A**) and TSD of *Acinetobacter* phage AM24 (PDB ID: 5W5P) (**B**) colored in gray. Two orientations are related by a rotation on 90° about vertical axis.

The comparative analysis demonstrated that the APK09_gp48 structure is similar to the TSD from *Acinetobacter* phage AM24 (AM24_gp50, PDB ID: 5W5P) with a corresponding RMSD of 1.3Å. Both TSDs are specific to the same CPS. The superposition of AM24_gp50 to APK09_gp48 structures revealed high similarity. The major difference was found within the N-terminal domains, where main chains diverge to more than 10Å (Figure 12). 

### 2.5. Evaluation of the Antivirulence Potential of TSD APK09_gp48 in a G. mellonella larvae Model of A. baumannii Infection

The antivirulence potential of the recombinant TSDs was evaluated on the example of APK09_gp48. The antivirulence potential of the TSDs was evaluated on the example of APK09_gp48 using the *A. baumannii* B05-induced infection in the *G. mellonella* larvae model.

**Figure 13:**
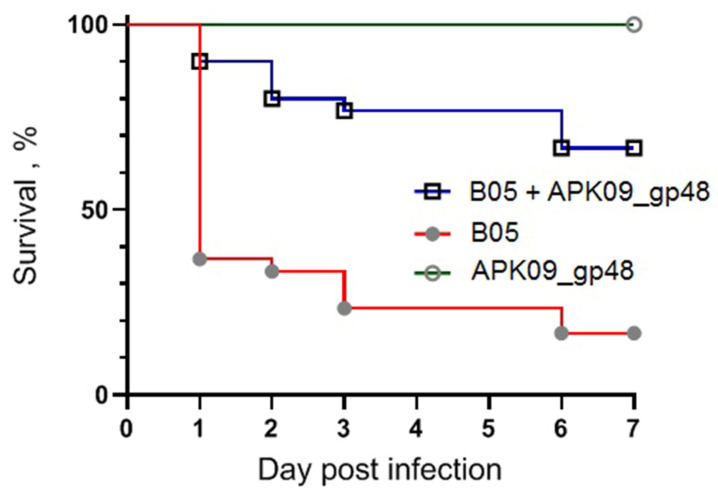
Kaplan–Meier survival curves following injection of *G. mellonella* larvae with *A. baumannii* B05 simultaneously with the depolymerase enzyme. Larvae (*n* = 30) were injected with either 3 × 10*^5^* CFU of *A. baumannii* B05 (red line) or the same bacteria simultaneously with APK09_gp48 (2µg/larva) (blue line). The experiment was controlled by the observation of uninfected larvae, larvae injected with saline solution, and larvae receiving depolymerase only (green lines). Survival for each control group was 100 %, so for simplicity, only group of depolymerase-injected larvae was included in the figure. Statistically significant differences in survival between larvae infected with bacteria only and larvae infected with bacteria simultaneously with APK09_gp48 were estimated by the log-rank (Mantel–Cox) test.

The infectious dose of 3 × 10^5^ CFU of *A. baumannii* B05, resulting gradual reduction in larval survival rates over the 7-day experiment, was selected. At the end of the 7-day follow-up period, 83.3% of the larvae died after inoculation with *A. baumannii* B05. At the same time, a single dose of enzyme APK09_gp48 injected together with the bacterial suspension significantly inhibited *A. baumannii*-induced death of *G. mellonella* larvae in a time-dependent manner (Figure 13). The injection of 2 µg of depolymerase with the infecting bacteria resulted in 66.7% survival of the larvae. Protection of the *G.mellonella* larvae by the depolymerase APK09_gp48 was found to be statistically significant (*p*-values < 0.0001).

No mortality of larvae was observed in the controls among uninfected larvae, larvae injected with saline solution, and larvae injected with depolymerase APK09_gp48 only.

## 3. Discussion

The polymorphism of the K loci in *A. baumannii* genomes results in variability of CPS structures and, consequently, determines a diversity of phage receptor-binding/recognizing proteins. Therefore, the identification of genes encoding TSDs in the genomes of capsular-specific *A. baumannii* phages, obtaining recombinant polysaccharide-depolymerizing enzymes, and studying their substrate specificity and mechanism of action expands our knowledge about the initial steps of the phage–host interaction. Moreover, the unique ability of specific phage-encoded depolymerases to recognize and degrade corresponding CPSs makes them an attractive and promising tool for combating pathogenic bacteria.

In this work, the TSDs encoded in the genomes of six novel Friunaviruses APK09, APK14, APK16, APK86, APK127v, APK128, and one previously described *Friunavirus* phage APK37.1 [11] were characterized and recombinantly produced. In the obtained trimeric structures of the TSDs APK09_gp48, APK14_gp49, and APK16_gp47, each chain contains a particle-binding N-terminal domain and a central β-helix domain, as well as a C-terminal domain (Figure 11) forming CPS-recognizing/degrading parts. All depolymerases studied were specific glycosidases that cleaved the corresponding CPSs by the hydrolytic mechanism with the production of monomers or/and oligomers (dimers and trimers) of the repeating K units (Table 2, Figure 4, Figure 5, Figure 6, Figure 7, Figure 8, Figure 9 and Figure 10). 

Four of the TSDs, namely, APK14_gp49, APK16_gp47, APK127v_gp47, and APK128_gp45, are the first reported phage-derived enzymes specific to the K14, K16, K127, and K128 *A. baumannii* CPSs, respectively. 

APK09_gp48 is specific to the K9 CPS, similar to the TSDs encoded in the genomes of Friunavirus vB_AbaP_B1 [2], Myoviruses AM24 [14], and BS46 [25]. The enzymatic activity of TSD BS46_gp47 has been studied earlier [26]. Both APK09_gp48 and BS46_gp47 cleaved *A. baumannii* B05 CPS by the β1→3 glycosidic linkage between the GlcNAc and GalNAcA residues of the neighboring K units. Digestion of the K9 CPS by both recombinant TSDs resulted in the formation of dimers and trimers of the repeating K unit. Therefore, despite the fact that depolymerases APK09_gp48 and BS46_gp47 do not share a high level of similarity at the amino acid level, the mechanism of their enzymatic activities is the same. Structure APK09_gp48 is also very similar to the structure of TSD AM24_gp50 from *Acinetobacter* phage AM24 (PDB ID: 5W5P), infecting the same bacterial host (Figure 12).

TSD APK86_gp49 is a structural protein of the phage APK86, initially isolated on the *A. baumannii* strain with the K86 CPS structure. BLASTp analysis revealed that the depolymerase was almost identical to TSD APK87_gp48 (QGK90498) of the previously characterized phage vB_AbaP_APK87 [4]. The structures of the K86 and K87 CPSs are presented by heptasaccharide K units distinguished from each other by only two monosaccharides. The linkages between the K86 and K87 units that are cleaved by the TSDs APK86_gp49 and APK87_gp48 are the same (Table 2; [4]). Therefore, the depolymerases are specific to both K86 and K87 CPSs. 

TSD APK37.1_gp49 was also found to digest the same linkages between K37 and K3-v1 units (Table 2; Figure 7A,B). Moreover, Friunavirus APK37.1 was shown to infect the *A. baumannii* strain having K116 [11] and K127 CPS structures with the same linkages between the oligosaccharide K units as in the K37/K3-v1 CPSs ([11]; Table 2). Interestingly, phage APK127v was found to be specific only to the K127 CPS-producing *A. baumannii* strain that carries prophage-encoded Wzy polymerase but was not able to infect strains with the similar K37/K3-v1 CPS structures, which requires further investigation. 

Over recent years, the antivirulence efficacy of several phage- and prophage-derived depolymerases specific to *A. baumannii* CPSs has been explored using a *G. mellonella* model [32,33,34]. In our experiments, recombinant TSD APK09_gp48 significantly inhibited *A. baumannii*-induced death of *G. mellonella* larvae. No mortality was observed in the control group of larvae injected with TSD APK09_gp48 only, demonstrating the safety of the depolymerase in this model. The results suggest that the depolymerase APK09_gp48 has the potential as therapeutics to prevent infections caused by *A. baumannii.*

## 4. Materials and Methods

### 4.1. Phage Isolation, Propagation, and Purification

Phages APK09, APK14, APK16, APK86, APK127v, and APK128 were isolated from sewage and river water samples collected in the Moscow region in 2018 on bacterial lawns of *A. baumannii* strains B05 (capsular type K9), AB5256 (K14), D4 (K16), MAR55-66 (K86), 36-1454 (K127), and KZ-1093 (K128). *A. baumannii* B05 was obtained from the State Collection of Pathogenic Microorganisms and Cell Cultures «SCPM-Obolensk» («SCPM-Obolensk» accession number B-7705). The other *A. baumannii* strains were kindly provided by the members of research groups from different countries (see Acknowledgements). For phage isolation, sewage and environmental samples were cleared by low-speed centrifugation at 7000× *g* for 15 min; then, the supernatants supplemented with LB medium were incubated in the presence of growing *A. baumannii* strains belonging to different capsular types overnight at 37 °C with shaking. After that, a portion of chloroform was added. Bacterial debris was pelleted by centrifugation at 7000× *g* for 30 min. Supernatants were then filtered through 0.45-µm-pore-size membrane filters (Merck Millipore, Cork, Ireland), and the purified filtrates were concentrated by ultracentrifugation at 85,000× *g* (Beckman SW50.1 Ti rotor, Beckman Coulter Inc., Brea, CA, USA) at 4 °C for 2 h. 

The search for lytic phages in the resultant concentrated preparations was conducted by a spot test, as well as plaque assay [35], on the lawns of the target *A. baumannii* strains. Single plaques with haloes found on the lawn of *A. baumannii* strains B05, AB5256, D4, MAR55-66, 36-1454, and KZ-1093 were picked up and suspended in the SM buffer (50 mM Tris-HCl pH 7.7, 8 mM MgSO_4_, 100 mM NaCl). The resulting solutions were replated three times to obtain pure phage stock.

The *A. baumannii* strains B05, AB5256, D4, MAR55-66, 36-1454, and KZ-1093 were used as bacterial hosts for further phage propagation. The process was executed using liquid culture of corresponding *A. baumannii* strains (OD_600_ = 0.3) at a multiplicity of infection (MOI) of 0.1 at 37 °C until lysis, and then chloroform was added. Bacterial debris was pelleted by centrifugation at 7000× *g* for 30 min. Phage particles were precipitated by polyethylene glycol (PEG) 8000 (added to a final concentration of 10% *w/v*) and 500 mM NaCl for 24 h at 4 °C. Further purification of the phages was performed by centrifugation in CsCl step gradient [36] at 100,000× *g* (Beckman SW50.1 Ti rotor, Beckman Coulter Inc., Brea, CA, USA) for 2 h; opalescent bands containing phages were collected, dialyzed against SM buffer, and stored at 4 °C.

### 4.2. Phage DNA Isolation and Sequencing 

Phage DNAs were isolated from concentrated and purified high titer phage stocks by phenol-chloroform method [36] after the incubation of the samples in 0.5% SDS and 50 µg/mL proteinase K at 65 °C for 20 min. The MiSeq platform and Nextera DNA library preparation kit (Illumina, San Diego, CA, USA) were used for phage genome sequencing. The generated reads were assembled de novo into a single contig using SPAdes v. 3.13 [37] with default parameters. The position and length of terminal repeats were identified by searching a region of greater coverage of sequencing reads in comparison to the average read depth along the whole genome of the phage. Physical termini were next verified directly by Sanger sequencing with outward-directed primers listed in Table S3.

### 4.3. Phage Genome Analysis

Potential open reading frames (ORFs) were identified using the RAST tool [38] and then manually inspected. The functions of genes were predicted using a BLAST search against the NR (non-redundant) database of the NCBI [39] and HHpred [40]. The tRNA coding regions were checked with tRNAscan-SE [41]. Comparative analysis of phage genome sequences was performed and visualized using Easyfig [42]. The intergenomic comparison was made with the Virus Intergenomic Distance Calculator (VIRIDIC) [43]. Phylogenetic analysis was performed using the amino acid sequences of major capsid proteins, large terminase subunits, head-to-tail connector proteins, DNA polymerases, and RNA polymerases encoded in related bacterial viruses deposited in the NCBI GenBank database. The alignments were made with MAFFT 7.48, with default settings, using the L-INS-i algorithm [44,45]. The phylogenetic tree was constructed with RAxML by rapid bootstrapping (bootstrap 1000) using the GAMMA LG F protein model [46].

### 4.4. Nucleotide Sequences Accession Numbers

The genome sequences of *A. baumannii* phages APK09, APK14, APK16, APK86, APK127v, and APK128 were deposited in GenBank under accession numbers MZ868724, MK089780, MZ868725, MZ936314, ON210142, and MW459163, respectively.

### 4.5. Cloning, Expression, and Purification of the Recombinant Depolymerases

The phage DNA sequences corresponding to the TSDs lacking N-terminal domains were amplified using PCR with the primers listed in Table S4 and then cloned into pTSL plasmid [47]. Expression vectors were transformed into chemically competent Escherichia coli B834(DE3) cells. Protein expression was performed in an LB medium supplemented with ampicillin at 100 µg/mL. Transformed cells were grown at 37 °C until the optical density reached a value of 0.6 at 600 nm. The medium was cooled to the temperature of 18 °C, followed by expression induction by the addition of isopropyl-1-thio-β-d-galactopyranoside (IPTG) to a final concentration of 1.0 mM. The cells were harvested by centrifugation at 4000× *g* for 20 min at 4 °C. Then, the cell pellets were resuspended in buffer A (20 mM Tris pH 8.0, 0.4 M NaCl) and sonicated (Virsonic, VirTis, France). The lysates were cleared by centrifugation at 13,000× *g* for 25 min and then loaded into 5-mL Ni^2+^-charged GE HisTrap columns (GE Healthcare Life Sciences, Chicago, IL, USA) equilibrated with buffer A. The proteins were eluted by a 0–200 mM imidazole step gradient in buffer A. His-tag and SlyD digestion was realized by incubation with tobacco etch virus (TEV) protease at a protease/protein ratio of 1/100 (wt/wt) overnight with simultaneous dialysis against 10 mM Tris pH 8.0 containing 1.0 mM 2-mercaptoethanol. Each cleaved protein was loaded onto a 5 mL SourceQ 15 (GE Healthcare Life Sciences, Chicago, IL, USA) column and eluted with a linear gradient of 0–600 mM NaCl in 20 mM Tris-HCl (pH 8.0). Protein-containing fractions were combined and concentrated using Sartorius ultrafiltration devices with a molecular mass weight cutoff of 50 kDa (Sartorius AG, Gottingen, Germany) to ∼10 mg/mL. Protein concentration was determined using the Bradford method with BSA as a standard.

### 4.6. Lawn Spot Assay

The enzymatic activity of TSDs was tested by spotting protein solutions onto the bacterial lawns of corresponding *A. baumannii* strains using the double-layer method [35]. For this, mixtures of *A. baumannii* host strain cultures grown in LB medium at 37 °C to OD_600_ of 0.3 with soft agar (LB broth supplemented with 0.6% agarose) were plated onto nutrient agar. Then, 10 μL aliquots of solutions containing N-deletion mutants of TSDs, and their tenfold dilutions were spotted on the soft agar lawns and incubated at 37 °C for 12–24 h.

### 4.7. Isolation, Purification, and Depolymerization of the CPSs by Recombinant Proteins

The *A. baumannii* strains B05 (K9), AB5256 (K14), D4 (K16), KZ-1101 (K37), AB5001 (K3-v1), MAR55-66 (K86), 36-1454 (K127), and KZ-1093 (K128) were cultivated in 2TY (16 g Bacto tryptone, 10 g Bacto yeast extract, 5 g NaCl) media overnight at 37 °C. Bacterial cells were harvested by centrifugation at 10,000× *g* for 20 min, washed with phosphate-buffered saline, suspended in aqueous 70% acetone, precipitated, and dried on air.

Samples of CPSs (K9, K14, K16, K37, K3-v1, K86, K127, K128) were isolated with phenol–water extraction protocol [48]. Dried *A. baumannii* cells were incubated with 45% aqueous phenol for 60 min at 70 °C. The extract was cooled and dialyzed. Insoluble contaminations were removed by centrifugation at 4000× *g* for 60 min. Aqueous 50% CCl_3_CO_2_H was added to CPS solutions in water at 4 °C; precipitates were removed by centrifugation at 4000× *g* for 60 min, and the supernatants were dialyzed with distilled water and freeze-dried. CPS preparations were heated with 2% HOAc at 100 °C for 2 h. Then, a lipid precipitate was removed by centrifugation at 12,000× *g* for 20 min. Purified CPS samples were isolated from the supernatant by gel permeation chromatography on an XK 26-mm (depth) by 70-cm (height) column (gel layer, 560 mm) (GE Healthcare Life Sciences, Chicago, IL, USA) of Sephadex G-50 Superfine (Amersham Biosciences, Uppsala, Sweden) in 0.05 M pyridinium acetate buffer, pH 4.5. 

Purified CPSs were solubilized at 20 mM TrisHCl pH 8.0 buffer, and 200–500 μg of recombinant TSDs were added for digestion. The reaction mixtures were incubated at 37 °C. CPS digestion products were fractionated by gel permeation chromatography on an XK 16-mm (depth) by 100-cm (height) column (gel layer, 800 mm) (GE Healthcare Life Sciences, Chicago, IL, USA) of Fractogel TSK HW-40S (Toyo Soda, Tokyo, Japan) in 1% acetic acid.

### 4.8. NMR Spectroscopy

Samples of purified CPSs were deuterium-exchanged and examined as solutions in 99,95% D_2_O on a Bruker Avance II 600 MHz spectrometer (Bruker, Karlsruhe, Germany). Sodium 3-trimethylsilylpropanoate-2,2,3,3-d_4_ (δ_H_ 0, δ_C_ −1.6) was used as an internal reference for calibration. Two-dimensional ^1^H-^1^H correlation spectroscopy (COSY), ^1^H-^1^H total correlation spectroscopy (TOCSY), ^1^H-^1^H rotating-frame nuclear Overhauser effect spectroscopy (ROESY), ^1^H-^13^C heteronuclear single-quantum coherence (HSQC), and ^1^H-^13^C heteronuclear multiple-bond correlation (HMBC) experiments were performed using standard Bruker software. Bruker TopSpin 2.1 program was used to acquire and process the NMR data. A spin-lock time of 60 ms and mixing time of 200 ms were used in ^1^H-^1^H TOCSY and ^1^H-^1^H ROESY experiments, respectively. A ^1^H-^13^C HMBC experiment was recorded with a 60 ms delay for the evolution of long-range couplings to optimize the spectrum for coupling constant J_H,C_ 8 Hz.

### 4.9. Crystallization, Data Collection, Processing, Structure Solution, and Refinement for the Recombinant TSDs

An initial crystallization screening of the recombinant TSDs was performed with a robotic crystallization system (Rigaku, Woodlands, TX, USA) and commercially available 96-well crystallization screens (Hampton Research and Anatrace, Aliso Viejo, CA, USA) at 20 °C using the sitting drop vapor diffusion method. The protein concentrations were as follows: APK09_gp48-10 mg/mL; APK14_gp49—6.0 mg/mL; APK16_gp47–8.5 mg/mL. Diffraction quality crystals were obtained by optimizing the initial crystallization condition using the vapor diffusion method (hanging drop) in 24-well VDX plates in the following conditions: APK09_gp48—2% PEG200, 100mM HEPES pH 7.5, 20% tacsimate pH 7.0; APK14_gp49—50mM CaCl_2_, 100mM Bis-tris pH 6.5, 30% PEG 550 MME; APK16_gp47–100 mM Tris pH 8.5, 2.0M ammonium sulfate 2.0 M.

Depolymerase crystals were briefly soaked in a mother liquor containing 20% glycerol (APK14_gp49 and APK16_gp47) or 100% parathone oil (APK09_gp48) immediately prior to diffraction data collection and flash-frozen in liquid nitrogen. The data were collected at 100K at BL41XU beamline (SPring8, Waco, Japan). The data were indexed, integrated, and scaled using the Dials [49] or XDS [50] programs (Table S5). The program Pointless [51] was used to suggest the corresponding space groups and detected twinning in the case of the APK16_gp47 structure.

The structures of APK14_gp49 and APK16_gp47 were solved at 2.12 Å and 2.59 Å, respectively, via the SAD method using SeMet derivatives. The location of the selene ions, solution of the phase ambiguity, density modification, and initial model building were made with the CRANK2 pipeline [52]. The high-resolution remote datasets for the native proteins were further used for refinement (Table S5). The structure of the APK09_gp48 was solved at 2.59 Å resolution by the molecular replacement method using the MOLREP program [53], and the corresponding domain models were prepared with AlphaFOLD [54].

The refinement of all structures was carried out using the REFMAC5 program of the CCP4 suite [55]. The visual inspection of electron density maps and the manual rebuilding of the model were carried out using the COOT interactive graphics program [56].

For APK09_gp48, the isotropic B-factor and hydrogen atoms in the fixed positions were used during refinement. In the final model, an asymmetric unit contained one copy of the protein (606 visible residues), 13 water molecules, and two PEG molecules from the crystallization solution. Seven N-terminal acids have no electron density.

For APK16_gp47, the refinement was performed using the detwinning option of Refmac5. The resolution was gradually increased to 1.50 Å using the isotropic B-factor and hydrogen atoms in the fixed positions. In the final model, an asymmetric unit contained one copy of the protein (632 visible residues), 78 water molecules, and one glycerol molecule from the cryo solution. Seven N-terminal acids have no electron density.

For APK14_gp49, the resolution was gradually increased to 1.55 Å using the isotropic B-factor and hydrogen atoms in fixed positions. In the final model, an asymmetric unit contained one copy of the protein (699 visible residues), 350 water molecules, one PEG molecule, one glycerol molecule, and one chloride ion from the crystallization solution. Nine N-terminal acids have no electron density. 

The visual inspection of the structures was carried out using the COOT program and the PyMOL Molecular Graphics System, Version 1.9.0.0 (Schrödinger, New York, NY, USA). The structure comparison and superposition were made using the PDBeFold program [57], while contacts were analyzed using the PDBePISA [58].

### 4.10. Galleria mellonella Larvae Infection Experiments

Greater Wax moth larvae (*G. mellonella*) were obtained from a laboratory culture maintained at State Research Center for Applied Microbiology and Biotechnology, Obolensk. Injections of larvae with *A. baumannii* cells and the estimation of the survival rate of infected larvae were performed as described [59]. Briefly, larvae were injected with 3 × 10^5^ CFU *A. baumannii* B05 cells, and bacteria at the same doses were administered together with the depolymerase APK09_gp48 (2 μg per larvae). Three control groups were used: uninfected larvae; larvae injected with saline solution; and larvae injected with depolymerase only. Infected larvae were incubated at 37 °C for 7 days, and mortality was recorded daily. Each test was performed in triplicate, with 10 larvae per trial. The GraphPad Prism software (GraphPad Software, Inc., San Diego, CA, USA) was used for statistical analysis performed for pairwise comparisons between larvae infected with bacteria only and larvae infected with bacteria simultaneously with depolymerase using log-rank (Mantel–Cox) test.

## Figures and Tables

**Table 1:** Characteristics of phage genomes encoding the TSDs.

Phage Name	Genome Length (bp*)*	DTR Length (bp*)*	G+C Content (%)	Total No of Genes	Genbank Accession Number	TSD Designation (ID)	Reference
APK09	41477	409	39.2	56	MZ868724	APK09_gp48 (UAW09804)	this work
APK14	41767	405	39.2	55	MK089780	APK14_gp49 (AYR04394)	this work
APK16	41135	357	39.4	54	MZ868725	APK16_gp47 (UAW09859)	this work
APK37.1	40966	339	39.2	56	MZ967493	APK37.1_gp49 (UAW07728)	[11]
APK86	41297	383	39.2	56	MZ936314	APK86_gp49 (UAW09972)	this work
APK127v	41380	422	39.2	53	ON210142	APK127v_gp47 (URQ05189)	this work
APK128	42013	428	39.2	52	MW459163	APK128_gp45 (QVD48888)	this work

**Table 2:** Cleavage of *A. baumannii* CPSs with specific TSDs.

Phage	TSDs	*A. baumannii* Host Strain	K Type	Linkage in CPS Cleaved by a Depolymerase	Depolymerization Products			
					Mono- mer	Di- mer	Tri- mer	Struc- tures
APK09	APK09_gp48	B05	K9	β-d-Glc*p*NAc-(1→3)-α-d-Gal*p*NAcA		**1**	**2**	Figure 4
APK14	APK14_gp49	AB5256	K14	α-d-Gal*p*NAc-(1→4)-β-d-Gal*p*NAc	**3**	**4**		Figure 5
APK16	APK16_gp47	D4	K16	β-d-Gal*p*-(1→3)-β-d-Gal*p*NAc	**5**			Figure 6
APK37.1	APK37.1_gp49	KZ-1101	K37	β-d-Gal*p*NAc-(1→3)-α-d-Gal*p*	**6**	**7**	**8**	Figure 7A
		AB5001	K3-v1	β-d-Gal*p*NAc-(1→3)-α-d-Gal*p*	**9**	**10**		Figure 7B
APK86	APK86_gp49	MAR55-66	K86	β-d-Gal*p*NAc-(1→3)-α-L-Rha*p*	**11**	**12**	**13**	Figure 8
APK127v	APK127v_gp47	36-1454	K127	β-d-Gal*p*NAc-(1→3)-α-d-Gal*p*	**14**			Figure 9
APK128	APK128_gp45	KZ-1093	K128	β-d-Gal*p*NAc-(1→4)-α-d-Gal*p*		**15**		Figure 10

## Data Availability

Annotated genomes of *A. baumannii* phages APK09, APK14, APK16, APK86, APK127v, and APK128 were deposited in GenBank under accession numbers MZ868724, MK089780, MZ868725, MZ936314, ON210142, and MW459163, respectively. Crystal structures of the TSDs APK16_gp47, APK09_gp48, and APK14_gp49 were deposited in the protein data bank (www.rcsb.org) with accession codes 8OPZ, 8OQ0 and 8OQ1, respectively.

## Supplementary Materials

The following supporting information can be downloaded at: https://www.mdpi.com/article/10.3390/ijms24109100/s1.
