# Supplementary material for: Friunavirus Phage-Encoded Depolymerases Specific to Different Capsular Types of Acinetobacter baumannii
Source: Int J Mol Sci. 2023 May 22;24(10):9100. doi: 10.3390/ijms24109100 (PMC10219094; doi:10.3390/ijms24109100)
Supplement: Supplementary file 1 [file ijms-24-09100-s001.zip › Supplementary Materials_revised.docx]

**Supplementary Materials**

**Figure S1.** VIRIDIC generated heatmap of 30 *Beijerinckvirinae* phages infecting *Acinetobacter baumannii*.


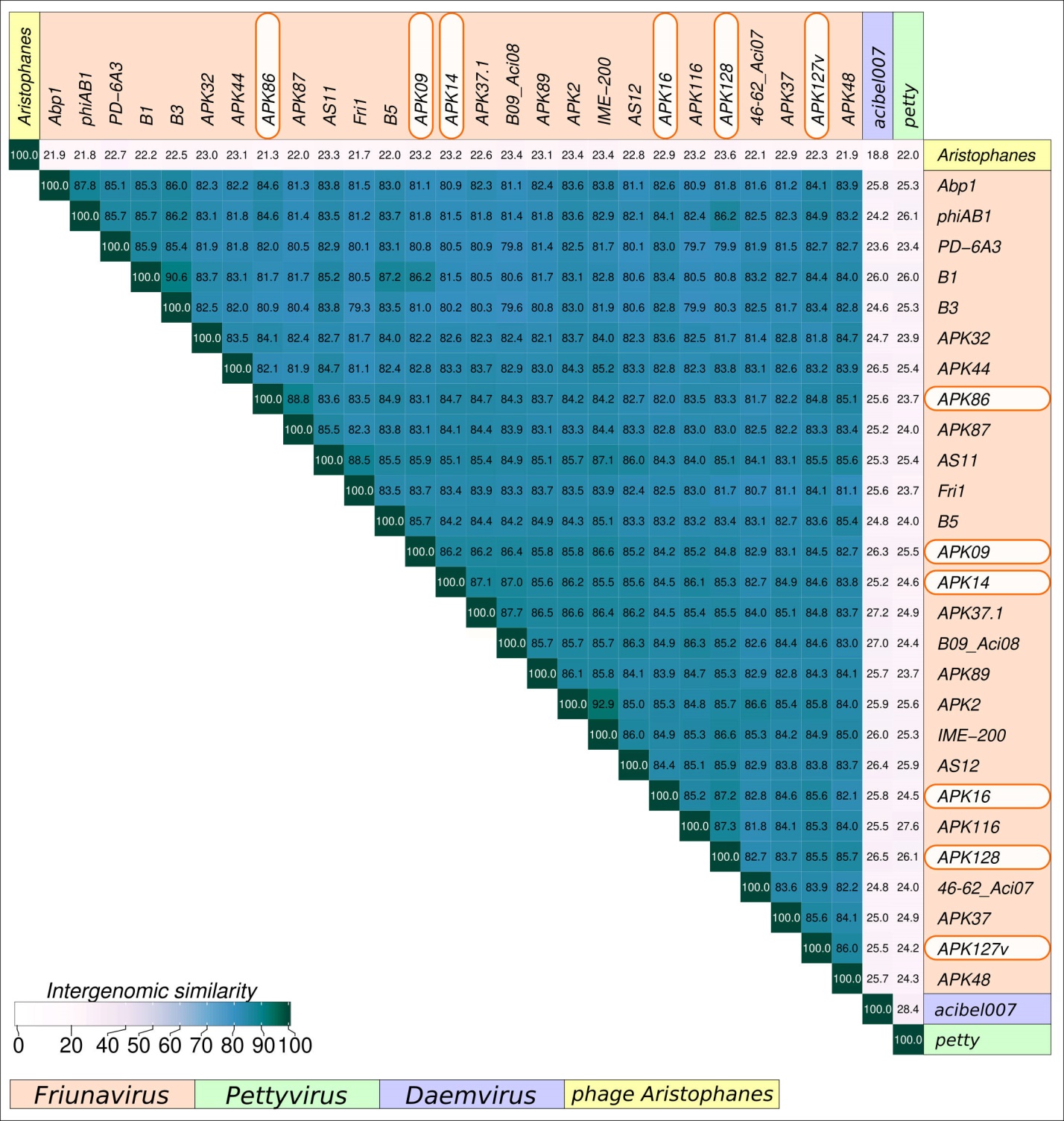


The color coding indicates the clustering of the phage genomes based on intergenomic similarity. The numbers represent the similarity values for each genome pair, rounded to the first decimal. The intergenomic similarity values of all novel phages are about 82-87% compare to the classified friunaviruses. These values are higher than the genus threshold of 70%.

**Table S1.** N-deletion TSD mutants.

| **TSDs** | **Genbank accession number** | **Full length**  **protein, aa** | **N-deletion TSD**  **mutants** | |
| --- | --- | --- | --- | --- |
|  |  |  | **Size, aa** | **Position** |
| APK09_gp48 | UAW09804 | 760 | 614 | 147-760 |
| APK14_gp49 | AYR04394 | 851 | 708 | 144-851 |
| APK16_gp47 | UAW09859 | 785 | 639 | 147-784 |
| APK37.1_gp49 | UAW07728 | 824 | 688 | 137-824 |
| APK86_gp49 | UAW09972 | 720 | 573 | 148-720 |
| APK127v_gp47 | URQ05189 | 663 | 517 | 147-663 |
| APK128_gp45 | QVD48888 | 878 | 739 | 140-878 |

Table S2. HR ESI-MS data of oligosaccharides 1 to 15 derived by depolymerization of *A. baumannii* CPSs with the specific TSDs.

| *A. baumannii* strain/  K type | Oligo-  saccha-  ride | Composition | Molecu-lar mass (Da) | Ion peak at m/z (exp./calc.) | | | | |
| --- | --- | --- | --- | --- | --- | --- | --- | --- |
|  |  |  |  | [M-H]^-^ | [M-2H]^2-^ | [M-3H]^3-^ | [M+Na]^+^ | [M+2Na]^2+^ |
| B05/K9 | 1 | HexN_6_HexNA_2_Ac_8_ | 1606.62 |  | 802.3092/802.3049 |  |  |  |
|  | 2 | HexN_9_HexNA_3_Ac_12_ |  |  | 1199.9654/1199.9665 |  |  |  |
| AB5256/K14 | 3 | Hex_3_HexN_2_Ac_2_ | 910.42 | 909.3211/909.3205 |  |  |  |  |
|  | 4 | Hex_6_HexN_4_Ac_4_ | 1802.64 | 1801.6370/1801.6377 |  |  |  |  |
| D4/K16 | 5 | HexHexNNon_1_Ac_3_ | 863.42 | 862.3276/862.3310 |  |  |  |  |
| KZ-1101/K37 | 6 | Hex_3_HexN_2_Ac_2_ |  |  |  |  | 933.3170/933.3170 |  |
|  | 7 | Hex_6_HexN_4_Ac_4_ |  |  |  |  | 1825.6318/1825.6342 |  |
|  | 8 | Hex_9_HexN_6_Ac_6_ |  |  |  |  |  | 1348.9904/1348.9900 |
| AB5001/  K3-v1 | 9 | Hex_2_HexN_1_HexA_2_N_3_N_4_O_1_Ac_3_  Hex_2_HexN_1_HexA_2_N_3_N_4_O_1_Ac_4_ | 803.29  845.29 | 802.2727/802.2735  844.2831/844.2841 |  |  |  |  |
|  | 10 | Hex_2_HexN_2_HexA_2_N_3_N_4_O_2_Ac_6_  Hex_2_HexN_2_HexA_2_N_3_N_4_O_2_Ac_7_  Hex_2_HexN_2_HexA_2_N_3_N_4_O_2_Ac_8_ |  |  | 793.2691/793.2682  814.2742/814.2735  835.2795/835.2788 |  |  |  |
| MAR55-66/  K86 | 11 | HexA_1_HexN_1_6dHex_5_ | 1129.42 | 1128.4142/1128.4199 |  |  |  |  |
|  | 12 | HexA_2_HexN_2_6dHex_10_ | 2236.80 |  | 1117.4000/1117.3990 |  |  |  |
|  | 13 | HexA_3_HexN_3_6dHex_15_ | 3346.17 |  |  | 1114.3989/1114.3972 |  |  |
| 36-1454/K127 | 14 | Hex_3_HexN_2_Ac_2_ | 910.32 |  |  |  | 933.3157/933.3170 |  |
| KZ-1093/K128 | 15 | Hex_6_HexN_4_Ac_4_ | 1826.72 |  |  |  | 1825.6321/1825.6342 |  |

**Table S3.** Oligonucleotide primers used in this study for verification of direct terminal repeats and physical termini of phage DNAs.

| **Primer** | **Sequence (5′–3′)** | **Phage** |
| --- | --- | --- |
| APK09_DTRR | cttgcaagttatagttgaatcca | APK09 |
| APK09_DTRL | gataaggagagaacgcataga | APK09 |
| APK14_DTRR | cttgcaagttatagttgaatcca | APK14 |
| APK14_DTRL | gataggtagatagaacgacataga | APK14 |
| APK16_DTRR | cttgcaagttatagttgaattca | APK16 |
| APK16_DTRL | agcatagaatgcatagatagga | APK16 |
| APK86_DTRR | cttgcaagttatagttaaatcca | APK86 |
| APK86_DTRL | aggcatgaactgaaagaacat | APK86 |
| APK127v_DTRR | cttgcaagttatagttgaatcca | APK127v |
| APK127v_DTRL | agagatgtatagagcgaatgcat | APK127v |
| APK128_DTRR | cttacaagttatagttgaactca | APK128 |
| APK128_DTRL | aggatgataagaggcaagtacta | APK128 |

**Table S4.** Oligonucleotide primers used in this study for cloning of phage TSDs.

| **Primer** | **Sequence (5′–3′)^a^** | **Restriction site for:** |
| --- | --- | --- |
| APK09_dep_F | ATA**GGATCC**GAGGAAGCTGCTCAAGTAGC | *Bam*HI |
| APK09_dep_R | ATA**CTCGAG**TTATGTGATAGTTAATAAGTTAGCA | *Xho*I |
| APK14_dep_F | ATA**GGATCC**GATGCTGCTGAGGAAG | *Bam*HI |
| APK14_dep_R | ATA**AAGCTT**AACACTTGAAATGTACGTATG | *Hind*III |
| APK16_dep_F | ATA**GGATCC**GAGGTAGCTGCTGCACAAAC | *Bam*HI |
| APK16_dep_R | ATA**CTCGAG**TTACACAGCAACCCAATTAG | *Xho*I |
| APK37.1_dep_F | ATA**GGATCC**caagctgctcaggaagctg | *Bam*HI |
| APK37.1_dep_R | ATA**AAGCTT**ATAAGACACTACTAACTGATTT | *Hind*III |
| APK86_dep_F | ATA**GGATCC**GAAGCTGCCGAAGATGCTT | *Bam*HI |
| APK86_dep_R | ATA**AAGCTT**AAATAAGTTTAATAAGTCCTCG | *Hind*III |
| APK127v_dep_F | ATA**GGATCC**GAGGAAGCTGCACAGACAAC | *Bam*HI |
| APK127v_dep_R | ATA**CTCGAG**TTAAACATTATAGTATGGCATCTTAT | *Xho*I |
| APK128_dep_F | ATA**GGATCC**CAAGAGGCTGCTAATGCAG | *Bam*HI |
| APK128_dep_R | ATA**CTCGAG**TTAATTTAGTCGAACATCAATC | *Xho*I |

^a^ Highlighted letters indicate restriction endonuclease recognition sites.

**Table S5.** Data collection and refinement statistics.

|  | **APK16_gp47** | | **APK09_gp48** | **APK14_gp49** | |
| --- | --- | --- | --- | --- | --- |
| **Data collection** | | | | | |
| **Diffraction source** | ESRF  (ID30B) | Spring8 (BL41XU) | ESRF  (ID30B) | ESRF  (ID30B) | Spring8  (BL41XU) |
| **Wavelength (Å)** | 0.97  (Se peak) | 0.70 (remote dataset) | 0.78 (remote dataset) | 0.97  (Se peak) | 1.0 (remote  dataset) |
| **Temperature (K)** | 100 | 100 | 100 | 100 | 100 |
| **Crystal-to-detector distance (mm)** | 197 | 250 | 563 | 418 | 220 |
| **Rotation range per image (°)** | 0.1 | 0.5 | 0.15 | 0.1 | 1.0 |
| **Total rotation range (°)** | 360 | 360 | 360 | 360 | 280 |
| **Space group** | H3 | H3 | H32 | P63 | P63 |
| ***a* (Å)**  ***b* (Å)**  ***c* (Å)** | 86.91  86.91  255.24 | 88.05  88.05  254.97 | 88.69  88.69  421.04 | 77.12  77.12  229.25 | 77.39  77.39  228.54 |
| **α (°)**  **β (°)**  **γ (°)** | 90.0  90.0  120.0 | 90.0  90.0  120.0 | 90.0  90.0  120.0 | 90.0  90.0  120.0 | 90.0  90.0  120.0 |
| **Resolution range (Å)** | 72.19–2.12 (2.18–2.12) | 85.0–1.50 (1.53–1.50) | 72.15–2.59  (2.70–2.59) | 66.79–2.66  (2.79–2.66) | 228.54–1.55  (1.58–1.55) |
| **Completeness (%)** | 99.2 (99.6) | 99.8 (98.5) | 99.1 (94.0) | 99.4 (97.1) | 100 (100) |
| **Average redundancy** | 3.5 (3.6) | 20.9 (20.9) | 18.5 (13.6) | 20.3 (19.9) | 15.3 (11.9) |
| **〈*I*/σ(*I*)〉** | 6.0 (1.7) | 4.7 (0.2) | 10.9 (1.7) | 15.2 (2.4) | 7.7 (0.2) |
| **Rpim (%)** | 7.8 (40.2) | 5.7 (132.1) | 8.1 (67.4) | 4.4 (35.1) | 3.9 (81.9) |
| **CC_1/2_** | 98.8 (84.4) | 99.6 (48.2) | 99.3 (30.2) | 99.4 (72.4) | 99.9 (43.1) |
| **Refinement** | | | | | |
| ***R_fact_* *(%)*** | 17.47 | | 18.87 | 19.96 | |
| ***R*_free._ *(%)*** | 21.47 | | 27.16 | 23.63 | |
| **RMS deviations** |  | |  |  | |
| Bonds (Å) | 0.02 | | 0.01 | 0.02 | |
| Angles (°) | 3.08 | | 2.38 | 2.10 | |
| **Ramachandran plot** |  | |  |  | |
| Most favoured (%) | 94.0 | | 89.6 | 96.1 | |
| Allowed (%) | 5.4 | | 8.3 | 3.7 | |
| **No. atoms** |  | |  |  | |
| Protein | 4733 | | 4588 | 5517 | |
| Water | 78 | | 9 | 350 | |
| Other ligands | 6 | | 14 | 14 | |
| **B-factors (Å^2^)** |  | |  |  | |
| Protein | 29.2 | | 59.0 | 28.1 | |
| Water | 31.7 | | 40.5 | 32.3 | |
| Other ligands | 27.8 | | 69.1 | 47.6 | |
| **PDB entry code** | 8OPZ | | 8OQ0 | 8OQ1 | |
